# Isosteric Substitution Enables Rational Design of Two‐Dimensional Energetic Crystals

**DOI:** 10.1002/advs.202523693

**Published:** 2026-04-15

**Authors:** Linyuan Wen, Wentong Tu, Tao Yu, Chao Chen, Zhixiang Zhang, Zhixiang Xie, Yingzhe Liu

**Affiliations:** ^1^ Xi'an Modern Chemistry Research Institute Xi'an P.R. China; ^2^ State Key Laboratory of Applied Organic Chemistry, College of Chemistry and Chemical Engineering Lanzhou University Lanzhou P.R. China; ^3^ National Key Laboratory of Energetic Materials Xi'an P.R. China; ^4^ State Key Laboratory of Fluorine & Nitrogen Chemicals Xi'an P.R. China; ^5^ Xi'an Key Laboratory of Liquid Crystal and Organic Photovoltaic Materials Xi'an P. R. China

**Keywords:** computation‐experiment loop, energetic materials, functional group isosterism, layer stacking, two‐dimensional materials

## Abstract

Two‐dimensional (2D) energetic crystals dissipate mechanical insult via interlayer slip, yet their molecular design space remains narrow. We introduce a functional group isosterism substitution strategy that expands the prevailing “NH_2–_C–C–NO_2_” motif to the aminofurazan unit, thereby enlarging accessible chemistries for 2D architectures. From an initial pool of 2832 candidates generated by database mining and high‐throughput enumeration, a tiered screening process incorporating virtual assessment and cross‐scale stability evaluation was employed. This process narrowed the focus to two high‐priority targets (**3** and **12**), which were subsequently synthesized and structurally confirmed to exhibit the desired layer crystal packing. Both synthesized materials demonstrate a highly desirable combination of high thermal stability, low mechanical sensitivity, and robust detonation performance. Most significantly, **12** achieves a superior balance by exceeding TATB in detonation velocity, detonation temperature, and heat of detonation while retaining comparable insensitivity, highlighting the strategy's capacity to balance energy and safety. The results close the computation‐experiment loop for 2D energetic crystals discovery and establish aminofurazan as a versatile energetic building block. More broadly, the strategy is generalizable to additional isosteres, such as aminotriazole derivatives, providing a principled blueprint for co‐optimizing energy and safety in next‐generation low‐sensitivity energetic materials.

## Graphical abstract

Isosteric substitution transforms a classical nitro–amine motif into two‐dimensional aminofurazans with performance beyond TATB.

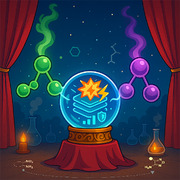

## 1 Introduction

**FIGURE 1:**
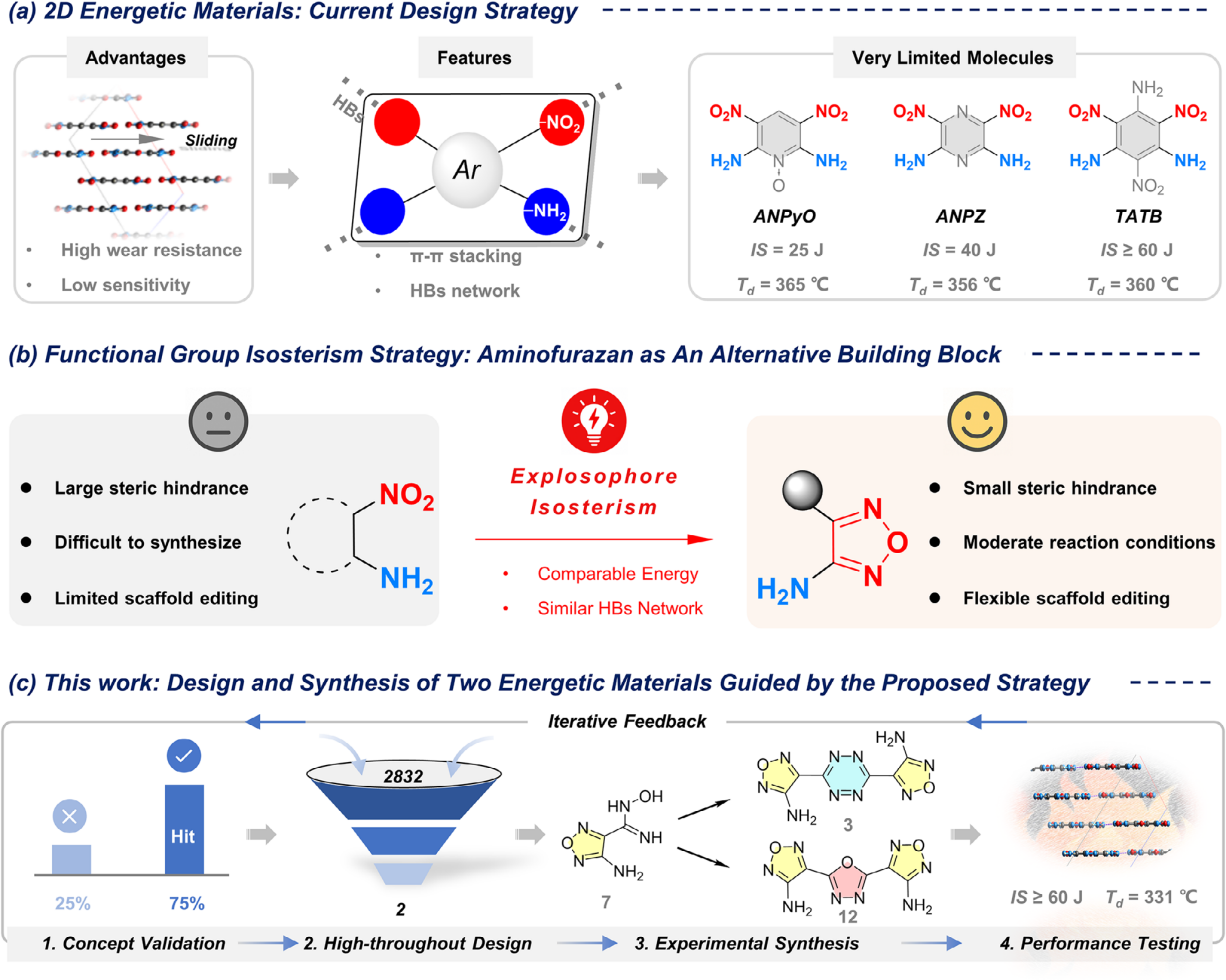
(a) The advantages of 2D materials, their detailed features, and current design strategies in energetic materials. (b) Functional groups isosterism strategy: from “NH_2_–C–C–NO_2_” to aminofurazan. (c) The workflow of the current work.

The isolation of a free‐standing, atomically thin crystal through mechanical exfoliation of graphite marked the beginning of the two‐dimensional materials era, with its outstanding electronic properties soon demonstrated by Geim and Novoselov's group in 2004 [1]. Two‐dimensional (2D) materials, exemplified by graphene, possess a unique layered structure (Figure 1A) that imparts exceptional physical and chemical properties, including high mechanical strength, low interlayer friction, and excellent electronic performance. These properties have enabled broad applications of 2D materials in optoelectronics, catalysis, adsorption, and energy storage, and energetic materials [2, 3, 4]. With the rapid advance of computational science, the design and synthesis of 2D materials have become a central focus of modern materials research and have seen substantial progress in recent years [5, 6]. In 2D energetic crystals, interlayer sliding under external stimuli suppresses hot spot formation and enhances safety, making them a leading direction for insensitive energetic materials [7]. Inorganic 2D materials (e.g., graphene; carbon nitride [8], and 2D covalent organic frameworks [9]) derive their extended planar architectures from covalent bonding, whereas organic energetic and other molecular crystals construct layered stacks via weaker interactions, notably hydrogen bonding and van der Waals forces. This fundamental difference renders the design of organic layered energetic crystals more demanding than that of inorganic 2D materials [10].

Over the past few years, meaningful progress has been made in the theoretical and experimental design of 2D energetic crystals [11, 12, 13, 14, 15]. New methodologies including Materials Genome approaches [16] and hydrogen‐bond pairing strategies [17] have provided fresh directions. Yet critical obstacles endure, most notably in the rational design of molecular building blocks. The “NH_2_–C–C–NO_2_” fragment is among the most widely used building blocks for 2D energetic crystals design, where it fosters in‐plane hydrogen‐bond networks [18] and interlayer π–π stacking. Despite this utility, designs anchored to this motif remain limited in chemical space and tunability, and only a handful of such crystals have been reported (e.g., 2,6‐diamino‐3,5‐dinitropyridine‐1‐oxide (ANPyO), 2,6‐diamino‐3,5‐dinitropyrazine (ANPZ), 1,3,5‐triamino‐2,4,6‐trinitrobenzene (TATB)). As shown in Figure 1B, it likely arises from: (1) substantial steric hindrance originated from the configuration hinders synthesis; (2) the use of concentrated nitric and sulfuric acids, leading to heavy post‐processing burdens and pollution; and (3) the requirement that the building block occupies multiple sites, leaving little editable space in the scaffold. Collectively, these factors have limited further progress in 2D energetic crystals.

To this end, a novel design strategy for 2D energetic crystals based on functional‐group isosterism replacement was proposed. The replacement motif must not only retain energetic characteristics comparable to the “NH_2_–C–C–NO_2_” building block, but also simultaneously supply hydrogen‐bond donors and acceptors, thereby enabling efficient intermolecular cooperativity within the crystal. Based on our previous work and related reports [19, 20, 21, 22, 23], the aminofurazan satisfies the above criteria while offering minimal steric hindrance, compatibility with mild reaction conditions, and limited site occupancy on the molecular scaffold. Accordingly, aminofurazan is proposed as a candidate building block for 2D energetic crystals.

To verify this hypothesis, a three‐stage workflow was established comprising concept validation, high‐throughput design, and experimental synthesis (Figure 1C). First, large‐scale database mining and statistical analysis were used to screen candidates that satisfy energy‐density and structural constraints, analyzing the applicability of aminofurazan in 2D energetic materials constructing. Next, guided by the functional group isosterism replacement template, 2832 tricyclic energetic candidates were high‐throughput generated via an enumeration method, and two targets (**3** and **12**) were selected after planarity screening and synthetic accessibility assessment. The targets were obtained through cyclization and oxidation, and the isolated yields was increased, exceeding 70% by optimization of reaction conditions. **3** (and **12**) both exhibit the outstanding properties: impact sensitivity 50 J (≥60 J), friction sensitivity ≥360 N (≥360 N), thermal decomposition temperature 285.2°C (331.0°C), and calculated detonation velocity 8339 m/s (8486 m/s), showing their strong potential as insensitive energetic materials. Besides, both the crystals of **3**∙DMSO and **12** display layered stacking, further validating the usefulness of the strategy in designing 2D energetic materials. Overall, the functional group isosterism strategy offers a new paradigm for co‐optimizing energy and safety in energetic materials, and provides fresh perspectives for 2D organic crystals design.

## 2 Results and Discussion

### 2.1 Concept Validation

**FIGURE 2:**
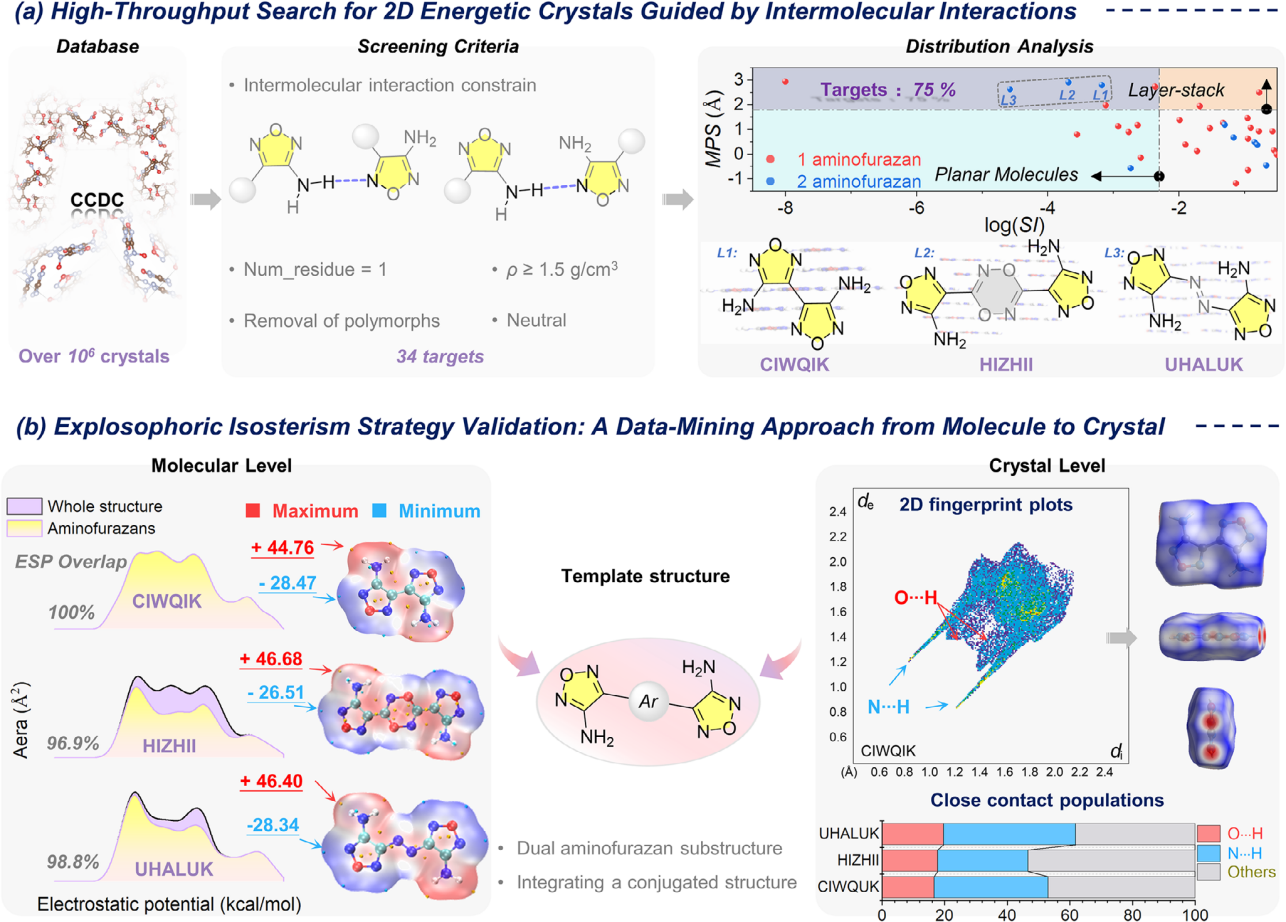
(a) Conceptual diagram of the screening process for 2D crystals derived from aminofurazan motifs, the blue dotted box highlighting the three 2D energetic crystals that were selected. (b) Elucidation of the pivotal role of the aminofurazan substructure in the rational design and assembly of 2D energetic crystals, with the left panel displaying electrostatic potential results and the right panel presenting Hirshfeld surface analysis results.

As shown in Figure 2A, to assess the potential of the aminofurazan motif for constructing 2D energetic crystals, five screening criteria was defined based on the Cambridge Structural Database (CSD): (1) presence of an aminofurazan unit with the corresponding intermolecular hydrogen bonds; (2) single component crystal; (3) crystal density ≥1.5 g/cm^3^; (4) neutral molecules; (5) exclusion of polymorphs, retaining structures measured near room temperature.

A total of 34 target structures were screened from over 10^6^ entries (Table S1). Structural distributions were analyzed using the maximum plane separation (*MPS*) [13] and shape index (*SI*) [24]. Layered stacking and molecular planarity were defined by *MPS* ≥ 1.8 Å and *SI* ≤ 0.005 respectively, and molecules were further grouped by the number of aminofurazan substructures. The *MPS* threshold was adopted from our previous study, while the *SI* cutoff was derived from representative layer‐stacked crystals, where the compound with refcode XIHLIL shows the highest *SI* value (*SI* = 0.0044) among known planar systems (Table S1). Accordingly, *SI* ≤ 0.005 was selected as the screening criterion. As shown in Figure 2A, no clear correlation between planarity and stack was observed for molecules bearing a single aminofurazan block. In contrast, for molecules containing two aminofurazan units, layered stacking was absent for *SI* >0.005 but present in 75% of planar cases (*SI* ≤0.005). These trends indicate that planar bis‐aminofurazan molecules preferentially form layered stack under hydrogen‐bond‐network constraints.

Based on the preceding result, the 3 screened layer‐stacked crystal (as seen in Figure S1) including CIWQIK, HIZHII, and UHALUK were analyzed through molecular electrostatic potential (ESP) and Hirshfeld surface analysis (Figure 2B). First, the aminofurazan substructures account for 100%, 96.9%, and 98.8% of the whole‐molecule ESP. More importantly, both the ESP maxima and minima localize on the aminofurazan units, enabling them to readily anchor the intralayer hydrogen‐bond network via adjacent aminofurazans and underscoring the importance of this motif.

From the 2D fingerprint plots and Hirshfeld surface (right panel, Figure 2B), the NH_2_ of the aminofurazan forms a short N∙∙∙H∙∙∙N contact to the nitrogen atom on the adjacent aminofurazan and additional N∙∙∙H∙∙∙O hydrogen bonds to the oxygen among the adjacent aminofurazan, indicating that this unit serves as the primary carriers of intralayer hydrogen bonding. Consistently, the close contact distribution shows that H∙∙∙O and H∙∙∙N interactions account for nearly 50% of contacts, further confirming the dominant contribution of the aminofurazan motif to intermolecular interaction. Therefore, it is inferred that two aminofurazan motifs dominate layer stacking through hydrogen‐bond network and electrostatic contributions, whereas the central fragments govern planarity and diversification. Thus, the molecule template is proposed as shown in Figure 2B, which features two aminofurazan groups that maintain an integrated 2D hydrogen‐bond network and a middle ring that enforces planarity and broadens the design space.

### 2.2 High‐Throughput Design

**FIGURE 3:**
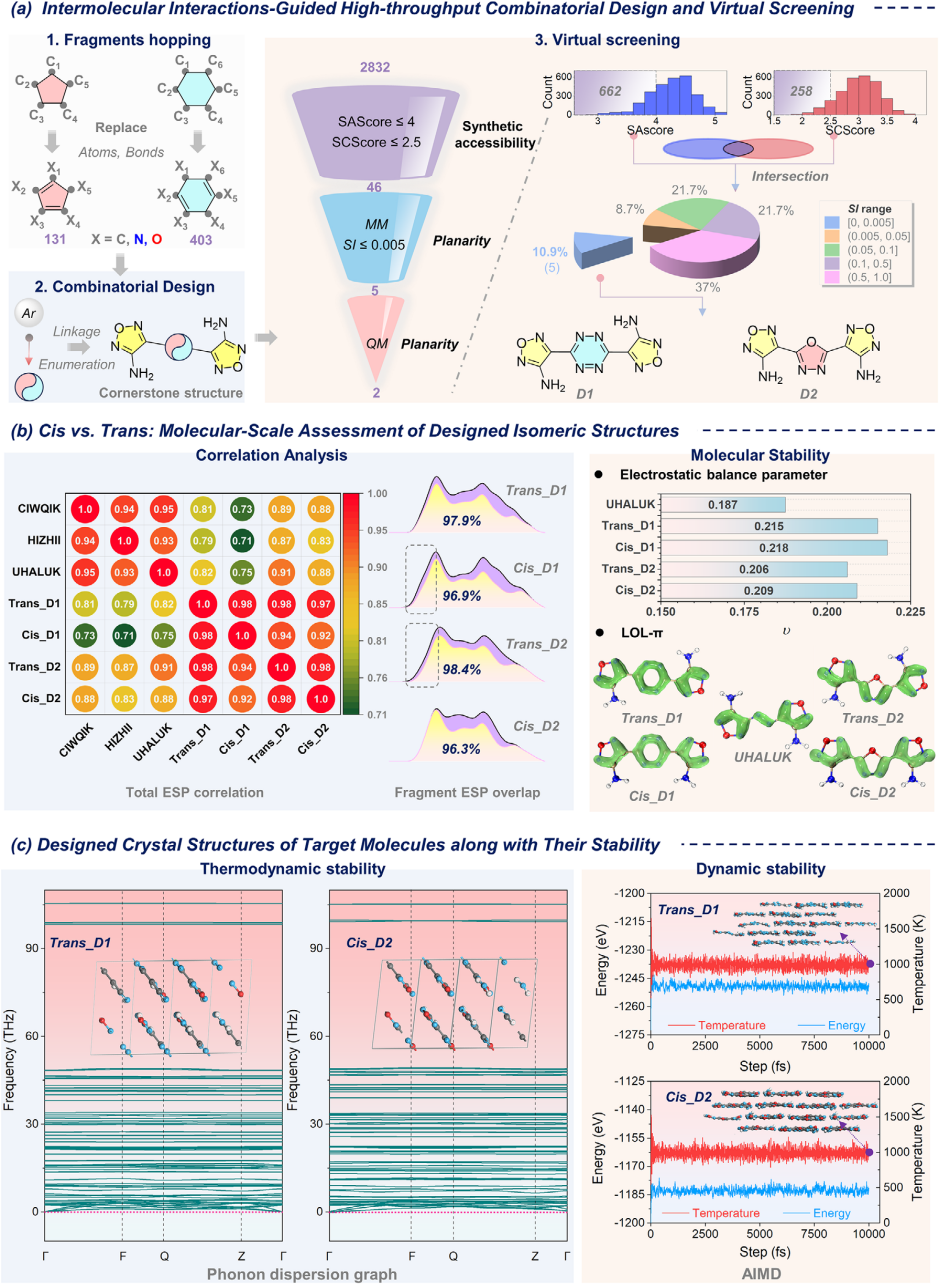
(a) High‐throughput design and screening of template based molecular candidates. (b) Molecular‐level comparison of cis and trans configurations. (c) Stability assessment of Trans_D1 and Cis_D2 crystals.

To expand the space of 2D energetic crystals, the high‐throughput design and screening workflow was implemented as shown in Figure 3A, including scaffold hopping, combinatorial design, and virtual screening:

(1) To align with the electrostatic and structural features of screened layered crystals, scaffold hopping was restricted to five‐ and six‐membered rings. The heteroatom set was limited to C, N, and O, and single/double‐bond patterns were randomized under valence saturation constraints, generating 131 five‐membered and 403 six‐membered ring scaffolds.
(2) Two sites within the ring structure were randomly selected and connected between the two aminofurazans, resulting in 2832 unique candidates following enumeration and deduplication.
(3) To rapidly screen accessible targets, two synthetic accessibility scoring models were employed including SAScore [25] and SCScore [26], and their results are shown in the blue and red bar graphs. Using the classic furazan‐based energetic molecule 3,4‐Dinitrofurazanfuroxccan (DNTF) [27] as a reference, molecules meeting the thresholds of SAScore≤4 (662 molecules) and SCScore≤2.5 (258 molecules) were retained. As previously reported, integrating these two metrics through an intersection approach significantly enhances the synthetic accessibility of the designed molecules [24]. Consequently, applying this filter reduced the candidate set to 46 molecules.

The molecular geometries were pre‐optimized using the MMFF94 force field in RDKit [28, 29], and the *SI* distribution (pie chart) indicates that only 10.9% meet *SI* ≤ 0.005 (selected structures in Figure S8). Besides, given the limited accuracy of force fields, the further optimization was performed at the M06‐2X/def2‐TZVP [30, 31], leading to the final selection of two planar candidates (D1 and D2).

Given the rotatable bonds in both D1 and D2, which could influence the structure packing mode in the crystal, the stability of their cis‐ and trans‐isomers at both the molecular and crystal levels were investigated. As depicted in Figure 3B, the ESP correlations are compared between the four designed molecules and three known layered stacking compounds. Since the three known layered stacking molecules are all trans structures, the ESP correlation of Cis_D1 and Cis_D2 are slightly lower than those of Trans_D1 and Trans_D2. Despite structural differences between the cis and trans isomers, the ESP for all designed molecules were found to exceed 0.7, indicating a strong electrostatic similarity to known layered stacking molecules. This suggests that the intermolecular interactions are expected to undergo minimal changes when arranged in the crystal lattice, thereby preserving electrostatic complementarity [32]. Despite a high degree (>95%) of spatial overlap in the ESP between the aminofurazan blocks and the complete molecular framework, the precise locations of the ESP extrema serve as a critical differentiator. In Cis_D1 and Trans_D2, the maxima and minima are displaced from the aminofurazan blocks, a deviation from the characteristic distribution observed in all three known layered stacking molecules. In contrast, Trans_D1 and Cis_D2 preserve the key feature of having their ESP extrema localized on the aminofurazan blocks. This alignment with the reference molecules strongly suggests that Trans_D1 and Cis_D2 are predisposed to maintain the necessary electrostatic complementarity for a layered stacking architecture, thereby promoting a stable crystalline packing akin to the known structures.

The stability of these four molecular structures were further assessed using electrostatic potential balance parameters (*ʋ*) and the localized orbital locator (LOL) function [33]. The results show that the *ʋ* for all four molecules exceed 0.2, slightly higher than the 0.187 value for UHALUK, suggesting that these molecules may be marginally less sensitive than UHALUK [34]. LOL analysis further reveals that the π electron distributions on the isosurface of the designed molecules closely resemble that of UHALUK. All structures exhibit extensive π electron conjugation and characteristic discontinuities at the N─O and C─O bonds. These shared electronic features, combined with their high *ʋ*, collectively suggest that the designed molecules possess impact sensitivity comparable to that of UHALUK [35].

In contrast to the conventional crystal structure search approach previously established by our group [36], the present study leverages the framework of crystal reverse engineering [10] to probe molecular packing and stability from a bottom‐up perspective. Starting from the layered stacking structure of HIZHII, which shares the same hydrogen bond network and three ring structure, the initial crystal structures of four molecules were constructed through atomic replacement and fragment rotation. The initial crystal structures and their corresponding isolated molecules were optimized using CP2K and Gaussian, respectively. As summarized in Table 1, Trans_D1 exhibits lower energy than Cis_D1 at the molecular and crystal levels, indicating that the trans isomer of D1 is more stable. In contrast, Trans_D2 has higher energy than Cis_D2 at both scales, suggesting that the cis isomer of D2 is more stable. Based on these results, the Trans_D1 and Cis_D2 crystals were selected for further investigation.

**TABLE 1:** The energy of optimized molecules and crystals.

Energy	Trans_D1	Cis_D1	Trans_D2	Cis_D2
**Molecule (Hartree)**	−928.983	−928.982	−894.753	−894.758
**Crystal (eV)**	−159.208	−159.020	−603.449	−604.384

To probe the lattice stability of the designed crystals, the phonon dispersion spectra for the most two promising candidates were evaluated, which was achieved by diagonalizing the force constant matrix. The phonon band structure was mapped along the high‐symmetry path Γ‐F‐Q‐Z‐Γ within the first Brillouin zone, providing a complete assessment of the vibrational properties and confirming the stability of the predicted crystal structures. As shown in Figure 3C, both Trans_D1 and Cis_D2 crystals exhibit imaginary phonon frequencies exclusively at the Γ point, with maximum values of 0.0049 THz and 0.1793 THz, which may result from numerical issues associated with small curvature potential surfaces during the geometry optimization process. Although these numerical artifacts could be reduced by employing larger supercells or tighter convergence criteria [37, 38], such strategies were deemed prohibitively costly for the present study. Similar imaginary frequency phenomena have been observed in phonon spectrum calculations of energetic materials such as HMX [39] and PETN [40]. Therefore, it can be concluded that the optimized Trans_D1 and Cis_D2 crystals are stable.

To further assess the dynamic stability of the designed crystals, first‐principles molecular dynamics simulations were performed with the results shown in Figure 3C. Both the energy and temperature trajectories for Trans_D1 and Cis_D2 remain stable throughout the simulation duration. Importantly, both crystals fully retain their original layered stacking motifs with no signs of phase transition or decomposition at the end of the simulation. These results collectively demonstrate that the Trans_D1 and Cis_D2 crystals possess robust thermal stability and are likely to maintain their structural integrity under practical conditions.

### 2.3 Experimental Synthesis

**FIGURE 4:**
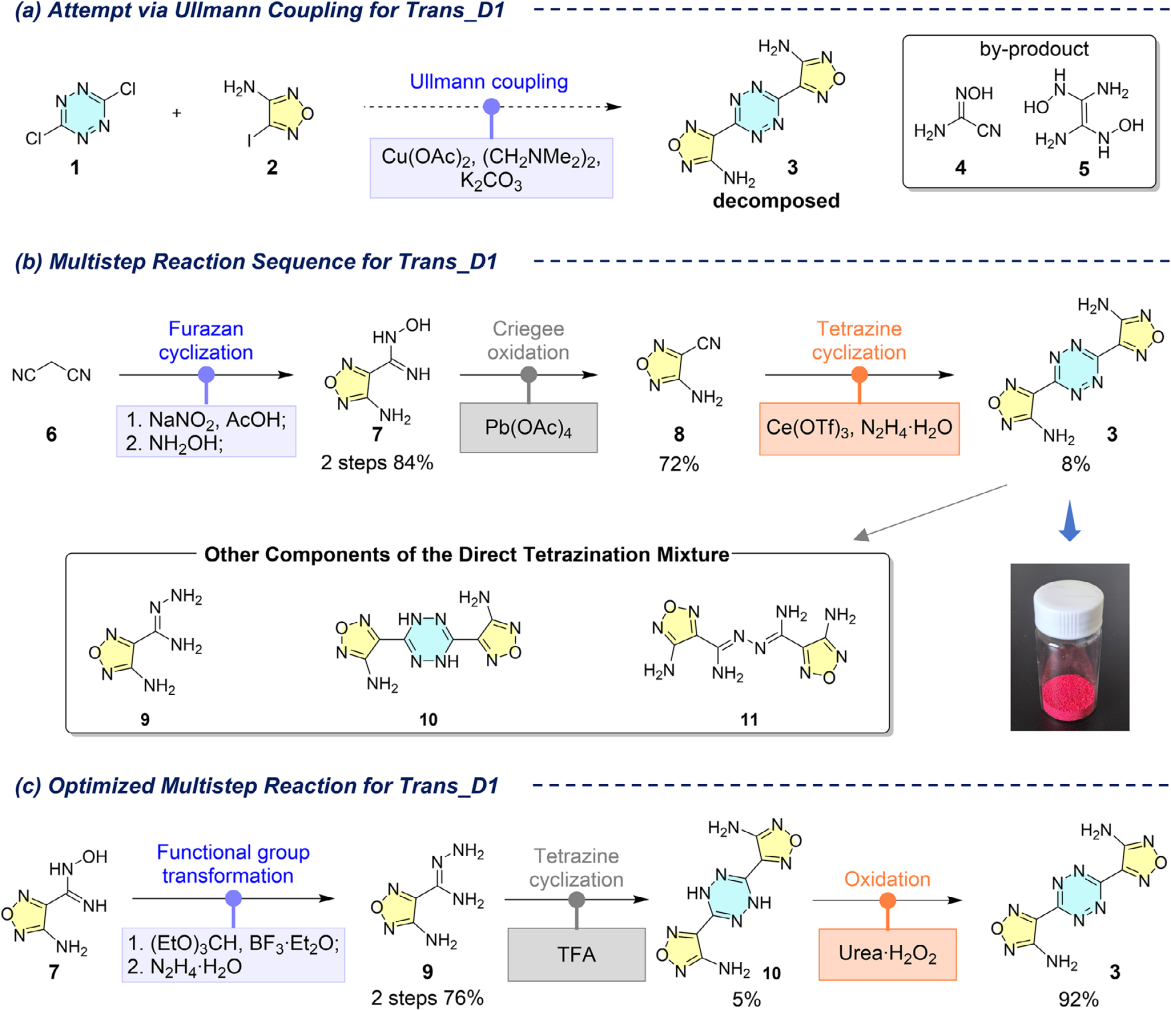
(a) Attempt via Ullmann coupling for Trans_D1. (b) Multistep reactions sequence for Trans_D1. (c) Optimized multistep reaction for Trans_D1.

In view of its stability and structural characteristics, experimental synthesis exploration was carried out focusing on the two structures Trans_D1 and Cis_D2 to verify the effectiveness of the functional group isosterism strategy in the design of 2D energetic crystals. As shown in Figure 4A, the Ullmann coupling reaction (**Path A**) between 3,6‐dichlorotetrazine (**1**) and 3‐amino‐4‐iodofurazan (**2**) was the initial substrate under copper catalysis with a diamine ligand. Contrary to expectations, the synthesis of **3** was not realized under the current catalytic conditions, presumably due to the facile copper‐catalyzed ring‐opening of 3‐amino‐4‐iodofurazan at elevated temperatures [41]. Instead, the reaction afforded hydroxyaminoimidomethyl cyanide (**4**) and 1,2‐bis(hydroxyamino)‐1,2‐ethylenediamine (**5**) as major products. Therefore, a new synthetic route needs to be designed to construct the tetrazine ring through an intermolecular cyclization reaction.

Because the direct coupling approach was unsuccessful, we turned to an alternative strategy in which the furazan ring would be constructed first, followed by the formation of the tetrazine ring to access the target compound. Accordingly, a new synthetic route was devised (Figure 4B), starting from readily available malononitrile (**6**), a cheap and widely used precursor for furazan synthesis. Employing a known furazan annulation protocol with NaNO_2_ and hydroxylamine furnished **7** in 84% yield. Subsequent oxidation of **7** with Pb(OAc)_4_ afforded 1‐amino‐2‐cyanofurazan (**8**) in 72% yield. From this intermediate, cyclocondensation with hydrazine hydrate under Ce(OTf)_3_ catalysis delivered **3** in 8% yield. Three byproducts were identified: 4‐aminofurazan‐3‐carbohydrazone (**9**, 21%), 3,6‐bis(aminofurazanyl)dihydrotetrazine (**10**, 27%), and bis(4‐aminofurazan‐3‐yl)azine (**11**, 34%). To our knowledge, acetamidrazone can undergo oxidation to form dihydrotetrazine [42]. Therefore, we propose that **3** is likely formed via the stepwise conversion of **9** and **10**. Consequently, optimizing the yield at each step should facilitate the efficient synthesis of **3** with high yields.

As seen in Figure 4C, a route was developed: **7** was reacted with triethyl orthoformate under Lewis acid catalysis and then hydrolyzed with hydrazine hydrate, to furnish **9** in 76% overall yield after two steps. The intermolecular cyclization used to construct the **10** was subsequently optimized (Table 2). Systematic optimization of the cyclization conditions revealed that the desired **10** was not formed below 80°C under acidic mediation. At 100°C with 10 mol% TFA, a 5% yield of **10** was obtained, alongside 51% of **11**. Importantly, increasing the TFA loading progressively suppressed the formation of **11** and enhanced the yield of **10**, with an optimal loading of 1.3 equivalents affording **10** in 75% yield. Finally, oxidation of the optimized intermediate **10** with urea hydrogen peroxide (UHP) cleanly delivered the target **3** in 92% yield.

**TABLE 2:** The optimized tetrazine cyclization reaction conditions for **10**.

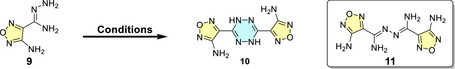				
Entry	Conditions	Solvent	Yield of 10 (%)	Yield of 11 (%)
1	AcOH (0.1 eq.), 50°C, 24 h	MeOH	0	64
2	AcOH (0.1 eq.), 50°C, 24 h	Dioxane	0	49
3	TFA (2.0 eq.), 80°C, 24 h	Dioxane	0	81
4	TFA (0.1 eq.), 100°C, 24 h	Dioxane	5	51
5	TFA (0.5 eq.), 100°C, 24 h	Dioxane	18	45
6	TFA (1.0 eq.), 100°C, 24 h	Dioxane	22	37
7	TFA (1.2 eq.), 100°C, 24 h	Dioxane	64	8
8	TFA (1.3 eq.), 100°C, 24 h	Dioxane	75	12
9	TFA (1.4 eq.), 100°C, 24 h	Dioxane	53	0

Given the complexity of synthesizing **3**, a direct route to **12** was envisioned through tetrazine transformation which would preserve the aminofurazan structures on both sides and avoid de novo synthesis. Because tetrazines undergo inverse‐electron‐demand Diels–Alder reactions with denitrogenation and are susceptible to nucleophilic attack, it was reasoned that **12** could be obtained in one step via acid‐mediated oxidation of **3**. Notably, Shreeve's group obtained **12** from a chain precursor in oleum in 2021 without crystallographic characterization [43].

As depicted in Table 3, a systematic screening of oxidation conditions was undertaken to achieve the direct conversion of **3** to **12** under mild conditions. Initial attempted employing m‐CPBA in CHCl_3_, H_2_O_2_/NaOH in MeOH/THF, or UHP in CH_2_Cl_2_/trifluoroacetic anhydride (TFAA) furnished no detectable product. Several subsequent systems achieved moderate success: H_2_O_2_/Na_2_WO_4_ in H_2_SO_4_ or 1,4‐dioxane gave **12** in 39% and 40% yield, respectively (entries 5 and 7). H_2_O_2_/SeO_2_ in 1,4‐dioxane provided 10% yield (entry 6). While H_2_O_2_/NMO in 1,4‐dioxane at 50°C was ineffective (entry 8), inclusion of (NH_4_)_2_S_2_O_8_ as an additive under otherwise identical conditions improved the yield to 42% (entry 9). Ultimately, an optimized protocol was identified: employing 10 equiv of UHP in formic acid at 50°C for 12 h furnished **12** in 76% isolated yield (entry 4).

**TABLE 3:** The optimized oxidation reaction conditions for **12**.

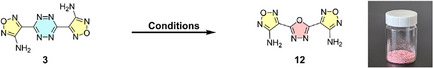			
Entry	Conditions	Solvent	Yield of 12 (%)
1	m‐CPBA (2.0 eq.), 70°C, 15 h	CHCl_3_	N.R.
2	H_2_O_2_/NaOH (10 eq.), 0°C, 10 h	MeOH/THF	N.R.
3	Urea∙H_2_O_2_ (10 eq.), 50°C, 12 h	DCM/TFAA	N.R.
4	Urea∙H_2_O_2_ (10 eq.), 50°C, 12 h	HCOOH	76
5	H_2_O_2_/Na_2_WO_4_ (10 eq.), 50°C, 12 h	H_2_SO_4_	39
6	H_2_O_2_/SeO_2_ (15 eq.), 50°C, 24 h	Dioxane	10
7	H_2_O_2_/Na_2_WO_4_ (15 eq.), 50°C, 24 h	Dioxane	40
8	H_2_O_2_/NMO (15 eq.), 50°C, 24 h	Dioxane	N.R.
9	H_2_O_2_/(NH_4_)_2_S_2_O_8_(15 eq.), 50°C, 24 h	Dioxane	42

### 2.4 Performance Testing

**FIGURE 5:**
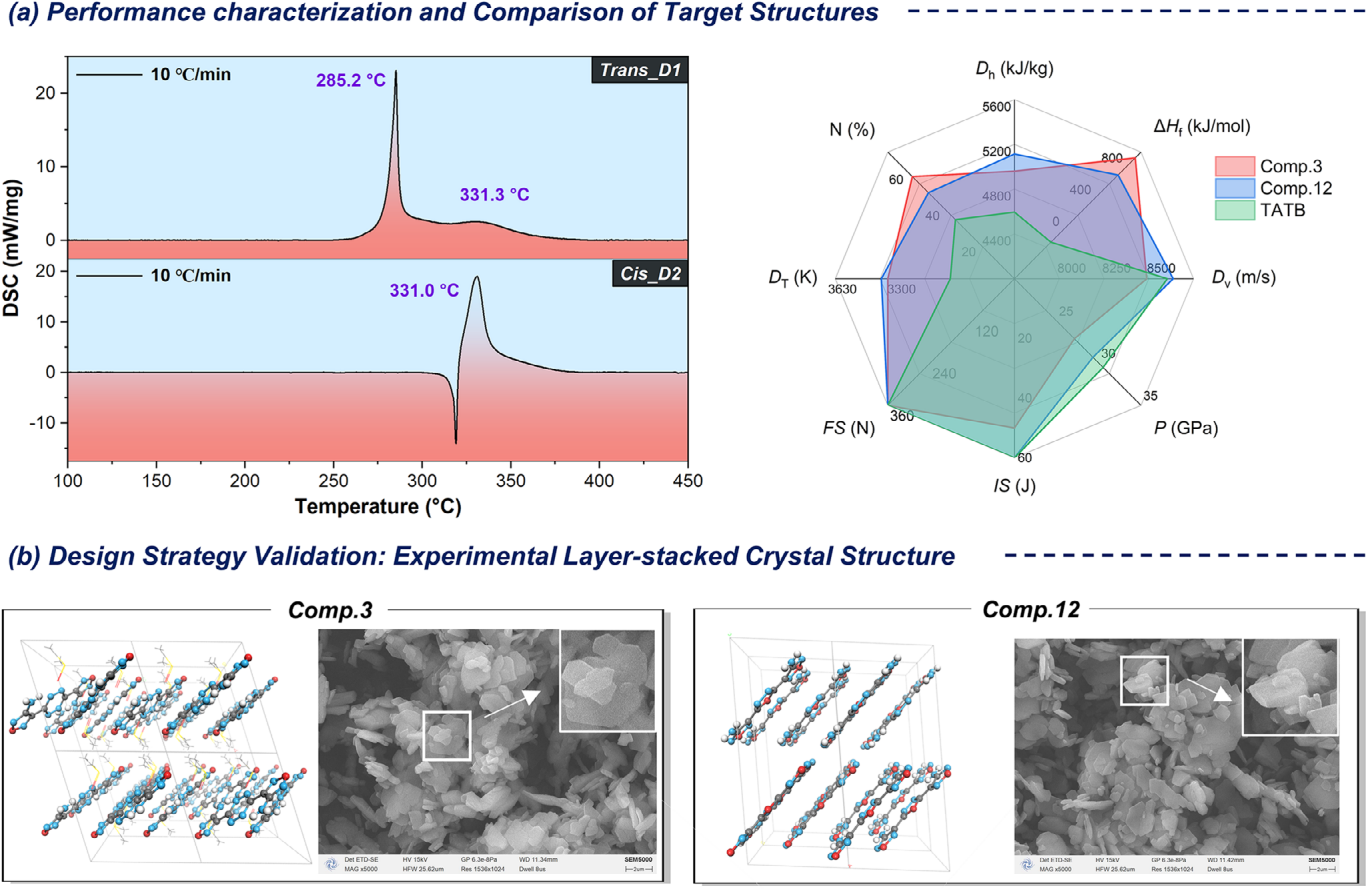
(a) DSC plots and performance comparison of **3** and **12**. (b) Crystal packing diagrams of **3**∙DMSO and **12**; SEM images of **3** and **12**, and the white frame is an enlarged image.

Following the successful synthesis, the thermal behaviors of **3** and **12** were characterized by DSC. Upon heating from 100 to 450°C, **3** displays two distinct exothermic events: a sharp exotherm peaking at 285.2°C, followed by a broader one at 331.3°C. In contrast, **12** melts below 325°C and subsequently undergoes a sharp exothermic decomposition at 331.0°C. The enhanced thermal resilience of **12** relative to **3** aligns well with the superior intrinsic stability of the 1,3,4‐oxadiazole over that of the tetrazine (Figure 5A). Gas pycnometer measurements yielded densities of 1.84 and 1.90 g/cm^3^ for **3** and **12** respectively (Table S2). Their calculated heats of formation were substantially higher (925.5 and 708.7 kJ/mol) than that of TATB (−139.5 kJ/mol), likely due to the higher nitrogen content of **3** (56.4%) and **12** (47.5%) relative to TATB (32.6%). Although their densities were lower, this was offset by the elevated heats of formation. Consequently, key detonation performance metrics, including detonation velocity (*D*_v_), detonation temperature (*D*_T_), and heat of detonation (*D*_h_), remain comparable to those of TATB. Furthermore, impact and friction sensitivity tests reveal notably low sensitivity (**3**: *IS* = 50 J, *FS* ≥ 360 N; **12**: *IS* ≥ 60 J, *FS* ≥ 360 N) as indicated in Table 4, in line with the design hypothesis that interlayer slip in 2D stacked crystals mitigates hot‐spot formation and lowers sensitivity.

**TABLE 4:** Physical and chemical properties of comp.**3** and **12** vs. TATB.

Comp.	*T*_d_ ^a^ (°C)	*ρ* (g/cm^3^)	Δ*H*_f_ ^c^ (kJ/mol)	*D*_v_ ^d^(m/s)	*P* ^e^(GPa)	*D*_h_ ^f^(kJ/kg)	*D*_T_ ^g^(K)	N %	*IS* ^h^(J)	*FS* ^i^(N)
3	285.2	1.84 ^b^	925.5	8339	27.1	4960	3457	56.4	50	≥360
12	331.0	1.90 ^b^	708.7	8486	29.3	5114	3493	47.5	≥60	≥360
TATB	360.0	1.94	−139.5	8453	30.5	4594	3108	32.6	≥60	≥360

^a^ Thermal decomposition temperature;

^b^ Density measured by gas pycnometer at 25°C;

^c^ Heat of formation (calculated using Gaussian 16);

^d^ Detonation velocity (calculated with Explo5 v6.05);

^e^ Detonation pressure (calculated with Explo5 v6.05);

^f^ Heat of Detonation (calculated with Explo5 v6.05);

^g^ Detonation temperature (calculated with Explo5 v6.05);

^h^ Impact sensitivity (BAM fall‐hammer tester method);

^i^ Friction sensitivity (BAM friction tester method).

To investigate whether **3** and **12** form layered stacking structures as expected, the crystals of **3**∙DMSO and **12** were obtained. As shown in Figure 5B, despite the absence of a solvent‐free form, the crystal structure of **3**·DMSO clearly exhibits a characteristic layered stacking motif. In this arrangement, the DMSO molecules are incorporated into the lattice without disrupting the overall layered architecture. In contrast, **12** crystallizes in a solvent‐free form and displays a well‐defined layered stacking structure, with an *MPS* of 3.01 Å. This pronounced and unperturbed stacking feature more reliably reflects the intrinsic structure–property relationship, supporting the effectiveness of the aminofurazan substitution strategy in promoting layered packing. The plate‐like crystallites of **3** and **12** observed via SEM originated from the synergistic effect of conjugated molecular structures and a directional hydrogen bond network, which promoted anisotropic crystal growth [44]. This 2D structure is pivotal for their low sensitivity, as the facile interlayer slip inherent to such packing dissipates mechanical energy and suppresses the nucleation of hot spot under stimuli. Consequently, the layered architectures of **3** and **12** provided a structural basis for their low sensitivity, unequivocally validating the functional group isosterism approach for constructing 2D energetic crystals.

## 3 Conclusion

This work establishes a functional group isosterism strategy for the rational design of 2D energetic crystals. By synergizing database mining, high‐throughput computational screening, and targeted synthesis, two predicted layered energetic materials (**3** and **12**) were successfully obtained, thereby completing a full design cycle from computation to experimental validation and confirming the effectiveness of our strategic approach. Notably, this study expands the building blocks for layered energetic crystals beyond the conventional NH_2_–C–C–NO_2_ motif to include the aminofurazan unit. This work not only identifies a novel energetic moiety for the design of 2D energetic crystals, thereby promoting the development of derivatives based on this building block, but also demonstrates that the functional group isosterism strategy can be extended to explore additional functional building blocks, such as aminotriazole. The successful implementation of the current strategy highlights the significant potential of functional group isosterism in optimizing the properties of energetic materials and provides both theoretical and experimental support for the future development of insensitive and stable energetic compounds.

## Conflicts of Interest

The authors declare no conflicts of interest.

## Supporting information




**Supporting File**: advs75279‐sup‐0001‐SuppMat.docx.

## Data Availability

The data that support the findings of this study are available from the corresponding author upon reasonable request.
