# Supplementary material for: Isosteric Substitution Enables Rational Design of Two‐Dimensional Energetic Crystals
Source: Adv Sci (Weinh). 2026 Apr 15;13(39):e23693. doi: 10.1002/advs.202523693 (PMC13334974; doi:10.1002/advs.202523693)
Supplement: Supplementary file 1 — Supporting File: advs75279‐sup‐0001‐SuppMat.docx. [file ADVS-13-e23693-s001.docx]

Supplementary Information

Isosteric Substitution Enables Rational Design

of Two-Dimensional Energetic Crystals

Linyuan Wen^a,†^, Wentong Tu^b,†^, Tao Yu^a, †^, Chao Chen^a^, Zhixiang Zhang^a^, Zhixiang Xie^b,*^, Yingzhe Liu^a,c,d,e,*^

[a] Dr. L. Wen, Dr. T. Yu, Dr. C. Chen, Dr. Z. Zhang, Prof. Y. Liu
Xi’an Modern Chemistry Research Institute
Xi’an 710065, P.R. China
E-mail: liuyz_204@ 163.com

[b] W. Tu, Prof. Z. Xie
State Key Laboratory of Applied Organic Chemistry, College of Chemistry and chemical Engineering, Lanzhou University
Lanzhou 730000, P.R. China
E-mail: xiezx@lzu.edu.cn

[c] Prof. Y. Liu
National Key Laboratory of Energetic Materials
Xi’an 710065, P.R. China

[d] Prof. Y. Liu
State Key Laboratory of Fluorine & Nitrogen Chemicals
Xi’an 710065, P.R. China

[e] Prof. Y. Liu
Xi’an Key Laboratory of Liquid Crystal and Organic Photovoltaic Materials
Xi’an 710065, P.R. China

[†] Both authors contributed equally to this work.

**Content**

**Experimental section**·········································································································1

**[Figure S1.](#_Toc211443703)** [The illustration of 2D bis-aminofurazan-based energetic crystals 4](#_Toc211443703)

**[Figure S2.](#_Toc211443704)** [The](#_Toc211443704) ^[1](#_Toc211443704)^[H NMR of Compound](#_Toc211443704) **[10](#_Toc211443704)** [(400 MHz, DMSO) 4](#_Toc211443704)

**[Figure S3.](#_Toc211443705)** [The](#_Toc211443705) ^[13](#_Toc211443705)^[C NMR of Compound](#_Toc211443705) **[10](#_Toc211443705)** [(101 MHz, DMSO) 5](#_Toc211443705)

**[Figure S4.](#_Toc211443706)** [The](#_Toc211443706) ^[1](#_Toc211443706)^[H NMR of Compound](#_Toc211443706) **[3](#_Toc211443706)** [(400 MHz, DMSO) 5](#_Toc211443706)

**[Figure S5.](#_Toc211443707)** [The](#_Toc211443707) ^[13](#_Toc211443707)^[C NMR of Compound](#_Toc211443707) **[3](#_Toc211443707)** [(101 MHz, DMSO) 6](#_Toc211443707)

**[Figure S6.](#_Toc211443708)** [The](#_Toc211443708) ^[1](#_Toc211443708)^[H NMR of Compound](#_Toc211443708) **[12](#_Toc211443708)** [(400 MHz, DMSO) 6](#_Toc211443708)

**[Figure S7.](#_Toc211443709)** [The](#_Toc211443709) ^[13](#_Toc211443709)^[C NMR of Compound](#_Toc211443709) **[12](#_Toc211443709)** [(101 MHz, DMSO) 7](#_Toc211443709)

**[Figure S8](#_Toc211443710)**[. 5 structures screened after MMFF94 optimization 7](#_Toc211443710)

**[Figure S9](#_Toc211443710)**[. The DSC/TG curves of compound 3 8](#_Toc211443710)

**[Table S1](#_Toc211443530)**[. The results of screened aminofurazan-based crystal 2](#_Toc211443530)

**[Table S2.](#_Toc211443531)** [The results of measured density 7](#_Toc211443531)

**Experimental Section**

**Computational Details**

The Gaussian 16 software package ^[1]^ was used for optimization and frequency calculations at the M06-2X/def2-TZVP level, and an ultrafine integration grid with D3 dispersion correction was used to calculate the formation enthalpy using the atomization energy method. Periodic density functional theory calculations were performed with CP2K ^[2]^. Exchange–correlation effects were described by the PBE generalized gradient approximation ^[3]^, and long-range dispersion was included via Grimme’s DFT-D3 correction ^[4]^. GTH–PBE norm-conserving pseudopotentials were used together with MOLOPT Gaussian basis sets, and the auxiliary plane-wave density grid employed CUTOFF = 600 Ry and REL_CUTOFF = 60 Ry. Geometry optimizations were carried out under periodic boundary conditions using a BFGS-type algorithm until the maximum force was < 1.0×10⁻⁴ Ha/Bohr and the electronic self-consistent field cycles converged to EPS_SCF = 2.0×10⁻⁹ Ha. Brillouin-zone sampling used Monkhorst–Pack meshes chosen to ensure a k-point spacing of ~0.02 Å⁻¹ along each reciprocal direction, with the same mesh retained for single-point force evaluations. Lattice-dynamical properties were obtained with Phonopy ^[5]^ using the finite-displacement method ^[6]^ on a 2×2×1 supercell and phonon dispersions were constructed from the resulting dynamical matrices. Dynamical stability was further assessed by Born–Oppenheimer molecular dynamics in the NVT ensemble (Nosé–Hoover thermostat) at 1000 K for 10 ps with a 1 fs time step, using identical electronic-structure settings throughout. The corresponding detonation performance was calculated by Explo 5 v6.05 software based on the BKW equation.

**Synthesis Details**

**General**. All reagents were purchased from Energy Chemical, Bidepharm, and Macklin as analytical grade and used as received. ^1^H and ^13^C NMR spectra were recorded on 400 MHz (Bruker Avance III 400) and 600 MHz (Avance NEO 600) instruments at 25 °C. Chemical shifts (δ) are given in ppm relative to residual solvent peaks (DMSO: δC = 39.52 ppm; δH = 2.50 ppm), and coupling constants (J) are in Hz. Decomposition temperatures were determined using a differential scanning calorimeter (Mettler Toledo DSC823e) at a scan rate of 10 °C·min⁻¹ in closed Al containers with a nitrogen flow of 10 mL·min⁻¹. Impact sensitivity (IS) and friction sensitivity (FS) measurements were performed according to BAM standards. All crystals were mounted on a MiteGen MicroMesh using a small amount of Cargille immersion oil. X-ray diffraction data were collected by XtaLAB Synergy-DW detector with graphite-monochromated CuKα radiation (λ = 1.54184). High-resolution mass spectra (HRMS) were acquired on a 4G mass spectrometer using electrospray ionization (ESI) with quadrupole time-of-flight (Q-TOF) analysis.

**Safety Statement**: All the compounds investigated are potentially explosive energetic materials. Although no hazards were observed during their preparation and handling, additional meticulous safety precautions are nonetheless necessary, including the use of grounded equipment, Kevlar gloves, Kevlar sleeves, a face shield, and earplugs.

3,6-bis(3-aminofurazanyl)dihydrotetrazine (**10**): Trifluoroacetic acid (3.5 g, 30.9 mmol) was slowly added to a solution of **9** (3.4 g, 24.11 mmol) in dioxane (34 mL). The mixture was then heated at reflux for 24 hours, during which time a yellow solid gradually formed. The suspension was filtered, and the cake was washed with 1,4-dioxane and EtOH. The combined filtrates were concentrated to dryness, the residue was triturated with H_2_O and EtOH, and the resulting solid was collected by filtration. The solids were combined and dried under reduced pressure to afford 10 as a pale yellow solid (4.5 g, 75%).

^1^H NMR (400 MHz, DMSO) δ 9.63 (s, 2H), 6.33 (s, 4H);

^13^C NMR (101 MHz, DMSO) δ 154.90, 139.12, 137.52;

IR (film) ν_max_ = 3451.90, 3305.21, 1649.42, 1622.61, 1605.64, 1530.58, 1426.50, 1379.69, 1124.43, 1082.19, 980.30, 920.09, 854.33, 771.43, 744.55, 711.72, 650.09, 570.46, 502.21 cm^–1^;

HRMS (ESI): m/z calcd for C_6_H_7_N_10_O_2_[M+H]^+^: 251.0748, found: 251.0751.

3,6-bis(3-aminofurazanyl)tetrazine (**3**): a 25 mL flask was charged with **10** (338 mg, 1.43 mmol), CH_2_Cl_2_ (10 mL), and UHP (15.0 equiv, 1.91 g). The suspension was stirred at 0 °C for 30 min, after which TFAA (10.0 equiv, 1.9 mL) was added dropwise. The mixture was maintained at 0 °C for 12 h and then warmed to room temperature and stirred for an additional 12 h, during which the precipitate turned bright red. The solid was collected by filtration, washed with CH_2_Cl_2_ (0.5 mL), and air dried to afford **3** as a bright-red powder (308.5 mg, 92%).

^1^H NMR (400 MHz, DMSO) δ 3.53 (s, 4H);

^13^C NMR (101 MHz, DMSO) δ 158.75, 156.18, 141.39;

IR (film) ν_max_ = 3447.12, 3316.83, 2970.53, 1615.20, 1539.18, 1445.63, 1405.72, 1297.59, 1255.45, 1140.91, 1115.03, 1080.94, 982.60, 933.23, 887.98, 853.88, 771.76, 746.50, 654.87, 615.11, 555.04, 451.73 cm^–1^;

HRMS (ESI): m/z calcd for C_6_H_5_N_10_O_2_ [M+H]^+^: 249.0591,found: 249.0593.

bis(3-aminofurazanyl)-1,3,4-oxadiazole (**12**): a 25 mL flask was charged with **3** (200 mg, 0.806 mmol) and formic acid (5 mL). After stirring at 0 °C for 30 min, the UHP was introduced in two equal portions: first 1.0 g (13.2 equiv), stirred for 15 min, followed by an additional 1.0 g (13.2 equiv). The mixture was then warmed to room temperature for 15 min and subsequently heated at 50 °C for 12 h, during which the bright-red precipitate turned pale pink. The solid was collected by filtration, washed with distilled water (0.5 mL), and air-dried to afford **12** as a pale pink powder (145.0 mg, 76%).

^1^H NMR (400 MHz, DMSO) δ 6.73 (s, 4H);

^13^C NMR (101 MHz, DMSO) δ 155.58, 155.38, 134.71;

IR (film) ν_max_ = 3442.79, 2999.74, 2914.75, 1654.46, 1437.85, 1407.64, 1313.93, 1030.32, 954.61, 704.30, 669.85, 608.18, 554.43, 492.85, 436.23 cm^–1^;

HRMS (ESI): m/z calcd for C_6_H_4_N_8_O_3_Na[M+Na]^+^: 259.0299, found: 259.0303.

**Crystal Growth**

DMSO was selected for single-crystal growth primarily because of the poor solubility of the compound **3** in most commonly used solvents, which greatly limited the possibility of obtaining suitable single crystals. Due to its stronger dissolving ability, DMSO was found to be more effective for dissolving the compound and thus more suitable for crystal growth.

**Statistical Analysis**

All experimental density measurements for compounds **3** and **12** were performed in quintuplicate (n = 5). Data are presented as mean ± standard deviation (SD). All theoretical calculations were performed using Gaussian 16 and do not involve further statistical treatment. Detailed statistical data for the density measurements are provided in **Table S2**.

**Table S1**. The results of screened aminofurazan-based crystal

| **Refcode** | **MPS (Å)** | ***SI*** | **Number of Fragments** | **Density (g/cm^3^)** |
| --- | --- | --- | --- | --- |
| BIRBAJ | 0.66 | 0.0685 | 2 | 1.681 |
| CIWQIK | 2.80 | 0.0007 | 2 | 1.789 |
| CUMLEE | 0.62 | 0.1136 | 1 | 1.762 |
| CUMLUU | -0.15 | 0.0026 | 1 | 1.650 |
| DAFPUV01 | 0.89 | 0.0017 | 1 | 1.863 |
| DAFRAD01 | 1.38 | 0.0103 | 1 | 1.603 |
| DOVCAT01 | 1.98 | 0.0008 | 1 | 1.731 |
| FAQZIK | 1.26 | 0.0474 | 1 | 1.546 |
| FEFXUM | 0.14 | 0.2846 | 1 | 1.767 |
| FIXQUC | -1.18 | 0.0744 | 1 | 1.576 |
| FIXRAJ | 0.12 | 0.0190 | 1 | 1.676 |
| FORMEG | 0.49 | 0.1437 | 2 | 1.847 |
| HIZHII | 2.90 | 0.0002 | 2 | 1.783 |
| JIRREK | 0.80 | 0.0003 | 1 | 1.795 |
| KASTOR | 1.17 | 0.0024 | 1 | 1.763 |
| LAQKOH | 2.94 | 0.0000 | 1 | 1.738 |
| LEWCUO | 0.37 | 0.1580 | 2 | 1.832 |
| MUXGIX | 1.12 | 0.0012 | 1 | 1.687 |
| NEZGUY | 1.46 | 0.1122 | 1 | 1.604 |
| NOQRAQ | 0.92 | 0.1657 | 1 | 1.528 |
| NOQRUK | 0.37 | 0.1233 | 1 | 1.642 |
| NUSTON | -0.02 | 0.3125 | 1 | 1.732 |
| OXAYAO | -0.57 | 0.0019 | 2 | 1.769 |
| QUSVOS | 1.04 | 0.0300 | 1 | 1.696 |
| REZMIW | 2.49 | 0.1690 | 1 | 1.737 |
| SAYFEH | -0.65 | 0.1133 | 1 | 1.924 |
| SOYREG | -0.47 | 0.2162 | 2 | 1.582 |
| UHALUK | 2.61 | 0.0000 | 2 | 1.767 |
| WAKNUS | 1.07 | 0.1248 | 1 | 1.573 |
| XIHLIL | 2.73 | 0.0044 | 1 | 1.904 |
| YUWYOH | 0.92 | 0.2677 | 1 | 1.699 |
| ZARJEJ | 1.19 | 0.0502 | 2 | 1.683 |
| ZIPSIC | 1.95 | 0.0211 | 1 | 1.692 |
| ZUDTEA | 0.39 | 0.0126 | 1 | 1.627 |


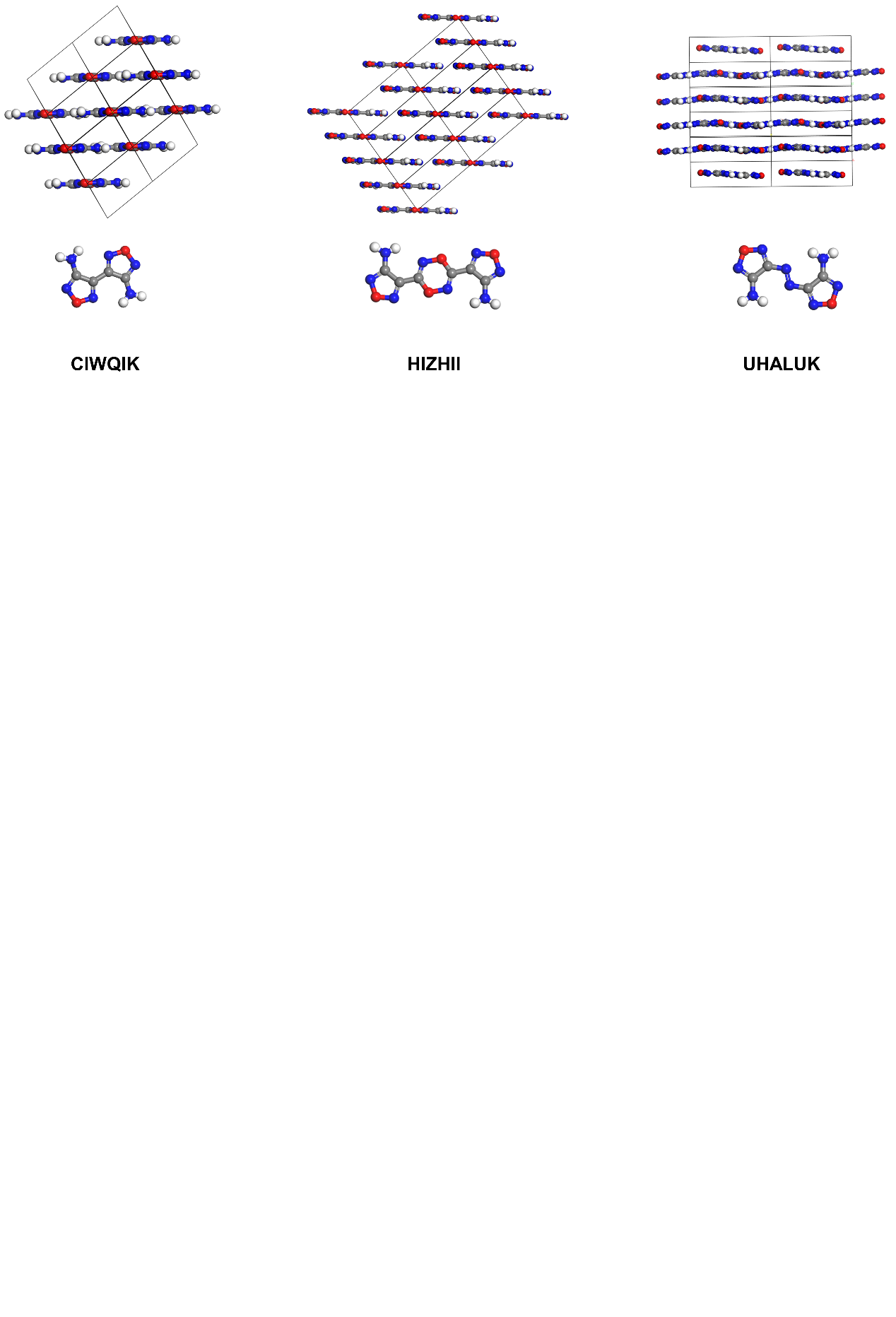


**Figure S1.** The illustration of 2D bis-aminofurazan-based energetic crystals


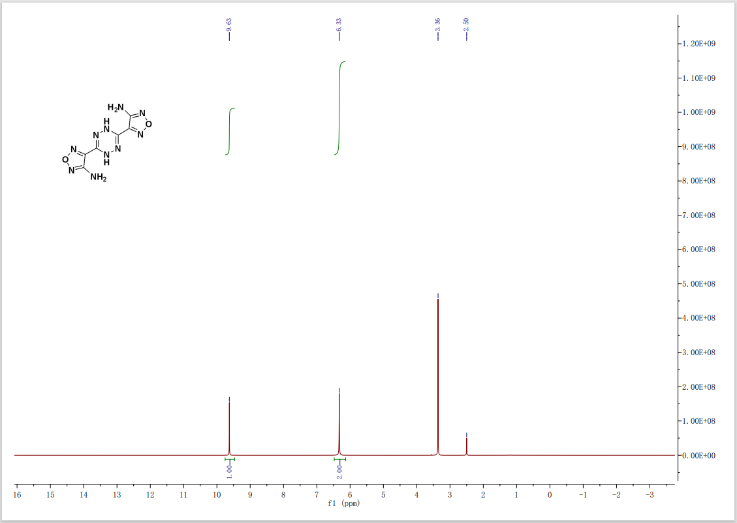


**Figure S2****.** The ^1^H NMR of Compound **10** (400 MHz, DMSO)


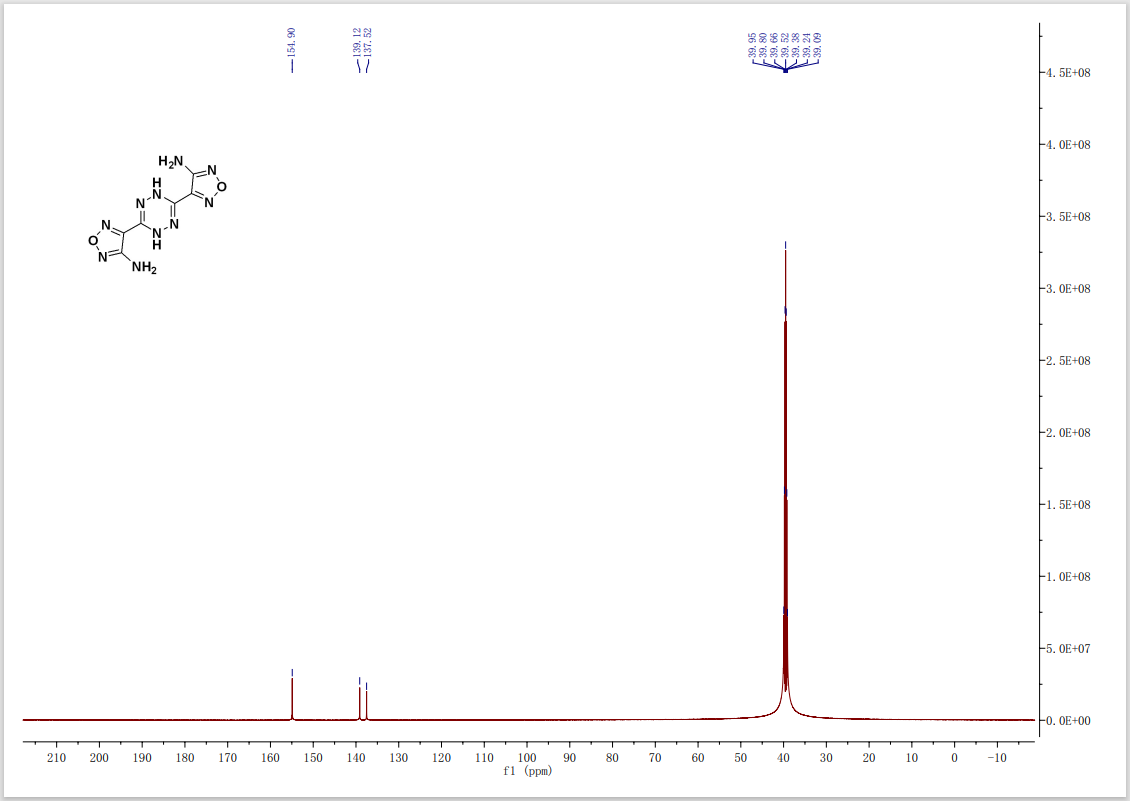


**Figure S3.** The ^13^C NMR of Compound **10** (101 MHz, DMSO)


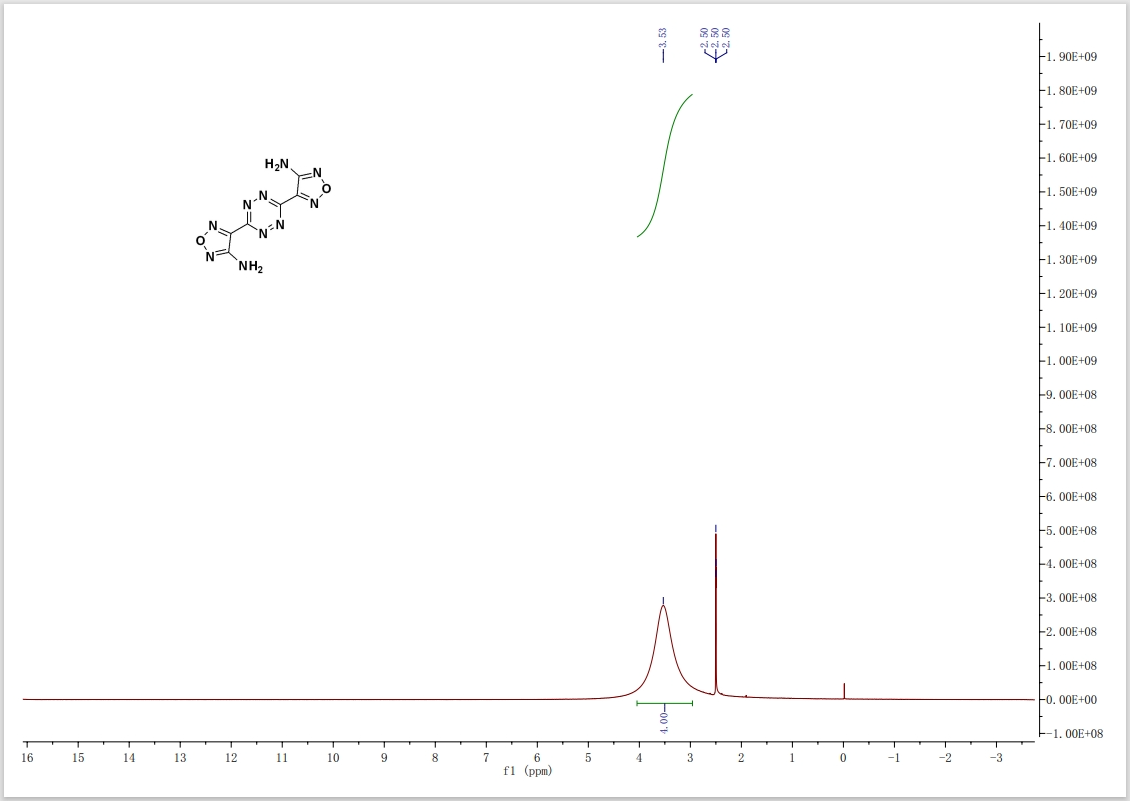


**Figure S4.** The ^1^H NMR of Compound **3** (400 MHz, DMSO)


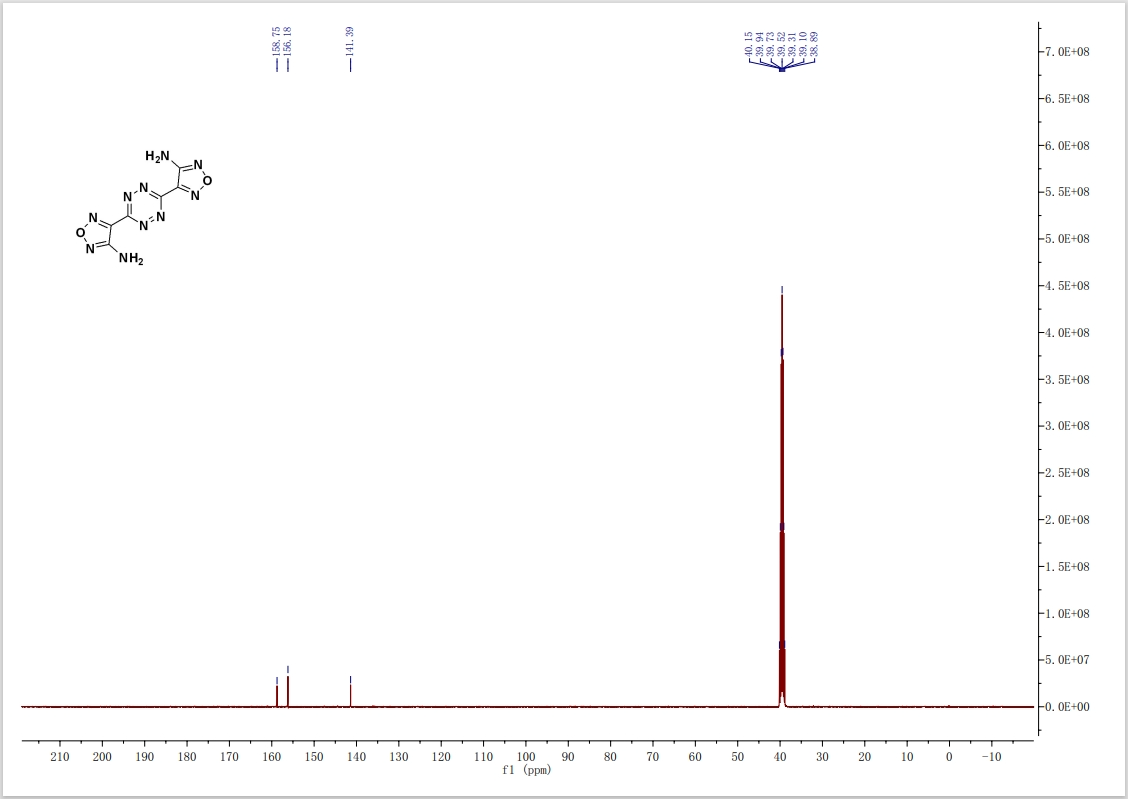


**Figure S5.** The ^13^C NMR of Compound **3** (101 MHz, DMSO)


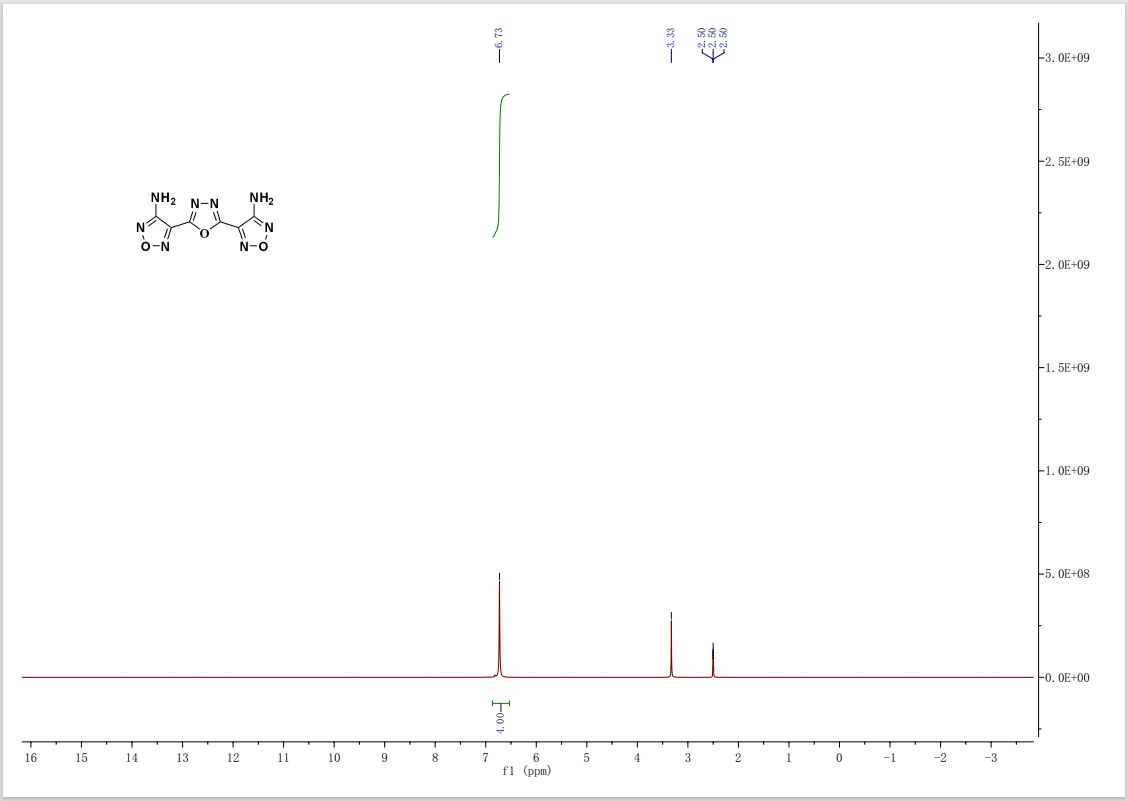


**Figure S6.** The ^1^H NMR of Compound **12** (400 MHz, DMSO)


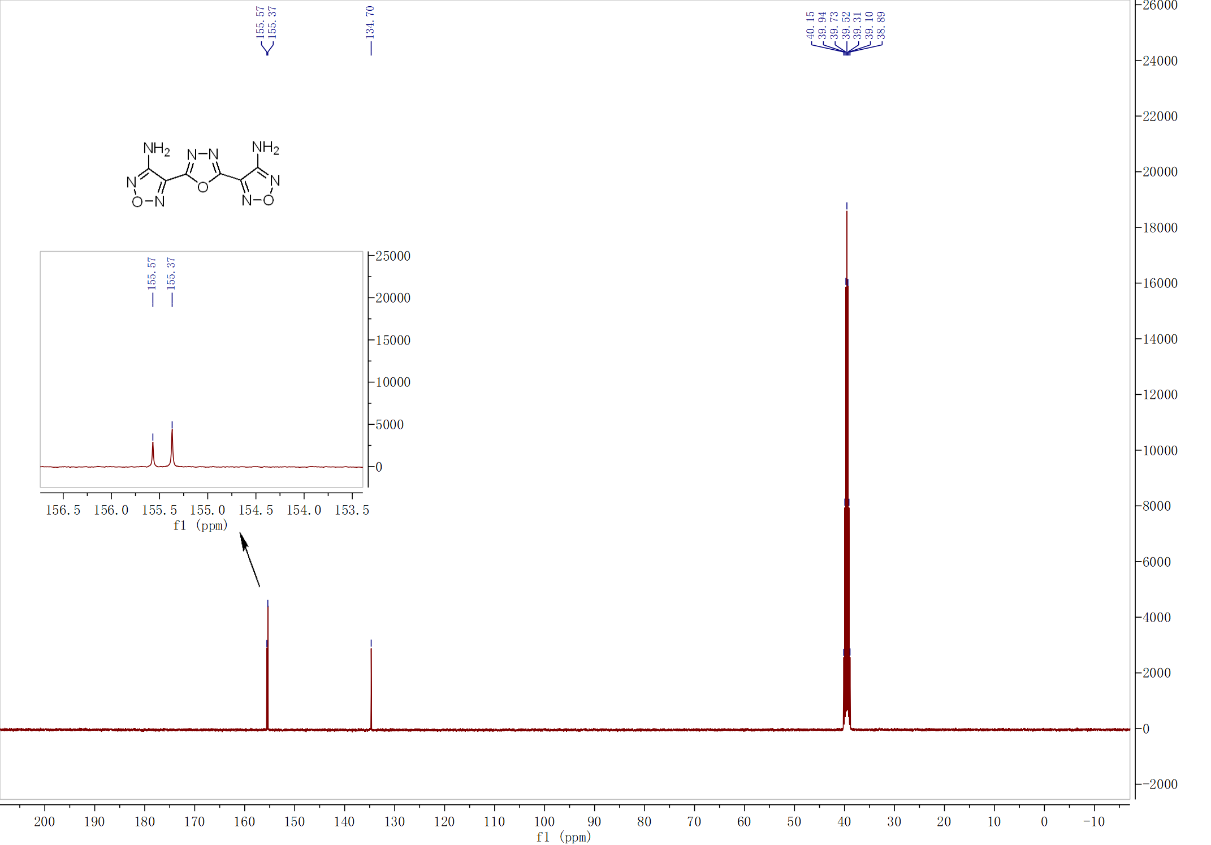


**Figure S7.** The ^13^C NMR of Compound **12** (101 MHz, DMSO)

**Table S2.** The results of measured density

| **Compound 3** | | **Compound 12** | |
| --- | --- | --- | --- |
| Test ID | Density (g/cm^3^) | Test ID | Density (g/cm^3^) |
| 1 | 1.8428 | 1 | 1.9020 |
| 2 | 1.8418 | 2 | 1.9040 |
| 3 | 1.8442 | 3 | 1.9043 |
| 4 | 1.8464 | 4 | 1.9057 |
| 5 | 1.8453 | 5 | 1.9056 |
| **mean value** | **1.8441** | **mean value** | **1.9043** |
| **SD value** | **0.0019** | **SD value** | **0.0015** |


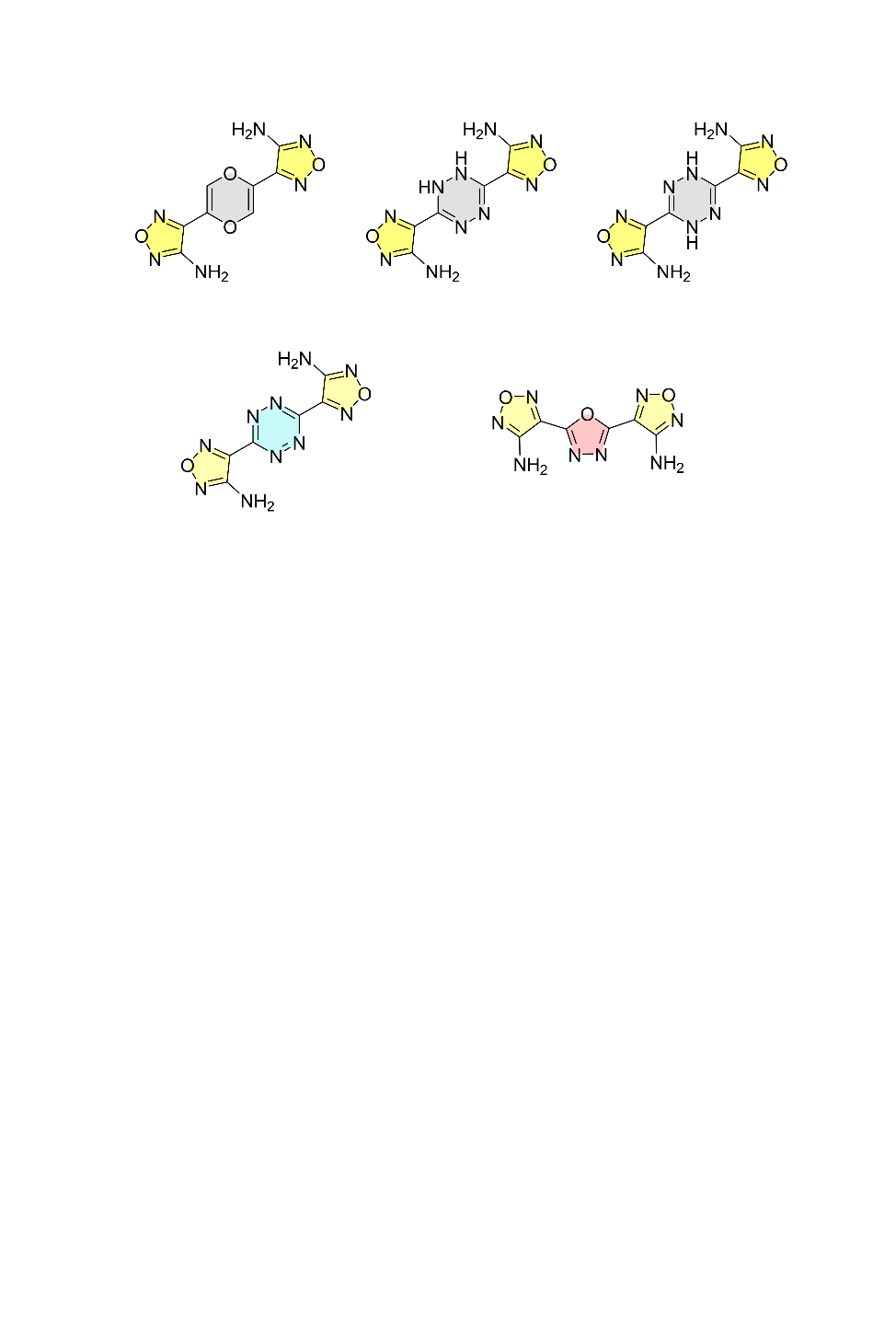


**Figure S8**. 5 structures screened after MMFF94 optimization


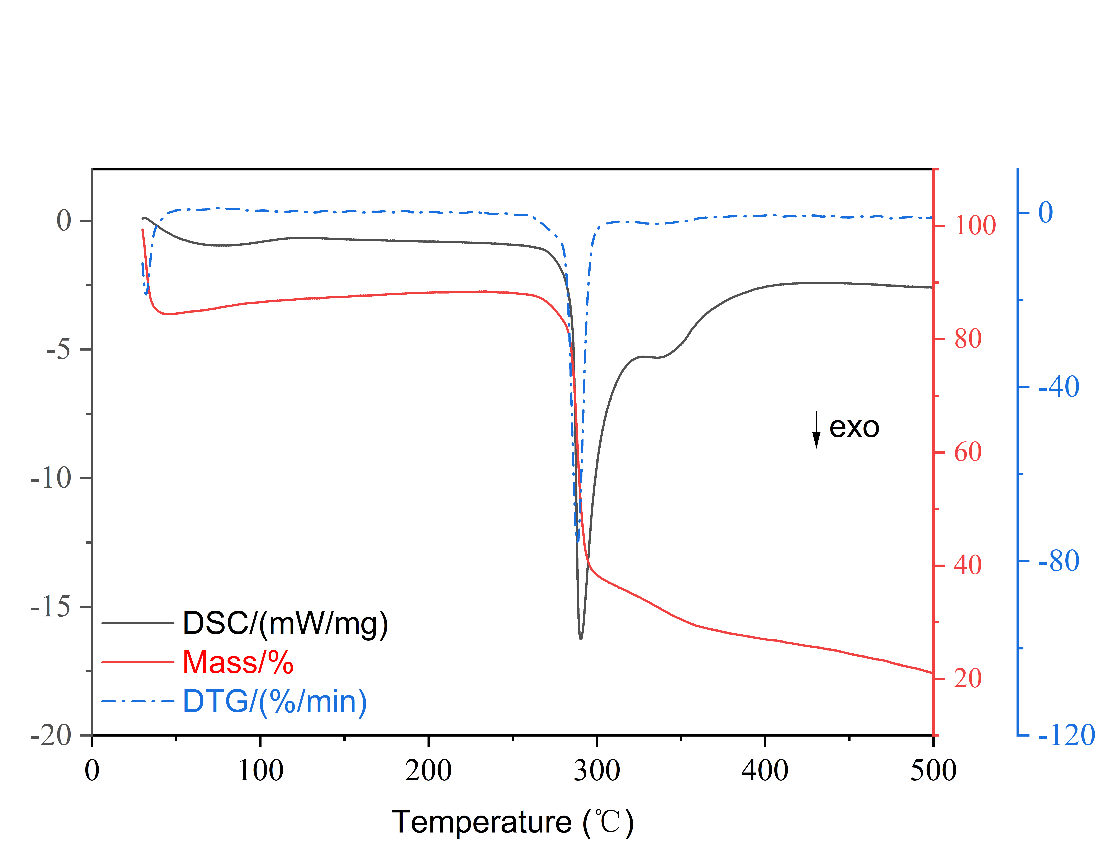


**Figure S9**. The DSC/TG curves of compound **3**

**Reference**

1. M. J. Frisch, G. W. Trucks, H. B. Schlegel, G. E. Scuseria, M. A. Robb, J. R. Cheeseman, G. Scalmani, V. Barone, G. A. Petersson, H. Nakatsuji, X. Li, M. Caricato, A. V. Marenich, J. Bloino, B. G. Janesko, R. Gomperts, B. Mennucci, H. P. Hratchian, J. V. Ortiz, A. F. Izmaylov, J. L. Sonnenberg, D. Williams-Young, F. Ding, F. Lipparini, F. Egidi, J. Goings, B. Peng, A. Petrone, T. Henderson, D. Ranasinghe, et al., Gaussian, Inc., Wallingford CT 2016, <https://gaussian.com/citation/.>
2. J. Hutter, M. Lannuzzi, F. Schiffmann, and J. VandeVondele, “CP2K: Atomistic Simulations of Condensed Matter Systems,” WIREs Computational Molecular Science 4, no. 1 (2014): 15–25.
3. J. P. Perdew, “Generalized Gradient Approximation Made Simple,” Physical Review Letters 77, no. 18 (1996): 3865–3868.
4. S. Grimme, J. Antony, S. Ehrlich, and H. Krieg, “A Consistent and Accurate Ab Initio Parametrization of Density Functional Dispersion Correction (DFT‐D) for the 94 Elements H‐Pu,” The Journal of Chemical Physics 132, no. 15 (2010): 154104.
5. A. Togo, “First‐Principles Calculations of the Ferroelastic Transition Between Rutile‐Type and CaCl_2_‐type SiO_2_ at High Pressures,” Physical Review B 78, no. 13 (2008): 134106.
6. D. Alfè, “PHON: A Program to Calculate Phonons Using the Small Displacement Method,” Computer Physics Communications 180, no. 12 (2009): 2622–2633.
